# Tyrosine‐Rich Peptides as a Platform for Assembly and Material Synthesis

**DOI:** 10.1002/advs.201801255

**Published:** 2018-11-15

**Authors:** Jaehun Lee, Misong Ju, Ouk Hyun Cho, Younghye Kim, Ki Tae Nam

**Affiliations:** ^1^ Department of Materials Science and Engineering Seoul National University Seoul 08826 Republic of Korea

**Keywords:** peptide assembly, peptide‐based hybrid materials, tyrosine, tyrosine‐rich peptides

## Abstract

The self‐assembly of biomolecules can provide a new approach for the design of functional systems with a diverse range of hierarchical nanoarchitectures and atomically defined structures. In this regard, peptides, particularly short peptides, are attractive building blocks because of their ease of establishing structure–property relationships, their productive synthesis, and the possibility of their hybridization with other motifs. Several assembling peptides, such as ionic‐complementary peptides, cyclic peptides, peptide amphiphiles, the Fmoc‐peptide, and aromatic dipeptides, are widely studied. Recently, studies on material synthesis and the application of tyrosine‐rich short peptide‐based systems have demonstrated that tyrosine units serve as not only excellent assembly motifs but also multifunctional templates. Tyrosine has a phenolic functional group that contributes to π–π interactions for conformation control and efficient charge transport by proton‐coupled electron‐transfer reactions in natural systems. Here, the critical roles of the tyrosine motif with respect to its electrochemical, chemical, and structural properties are discussed and recent discoveries and advances made in tyrosine‐rich short peptide systems from self‐assembled structures to peptide/inorganic hybrid materials are highlighted. A brief account of the opportunities in design optimization and the applications of tyrosine peptide‐based biomimetic materials is included.

## 1 Introduction

Proteins/peptides are small biological machines whose functions are wide ranging; their functions include molecular recognition, catalysis, replication, and mechanical responsiveness.^1^, ^2^, ^3^, ^4^ In addition to their biofunctionality, peptides are also used as precursors to encode and control the growth of inorganics in many living organisms. For example, organisms such as diatoms and marine sponges use self‐assembled peptides, proteins, and organic–inorganic suprastructures as templates to control nucleation and amorphous‐to‐crystal phase transformations, which leads to the generation of crystals with unusual shapes and complexity that have controlled sizes, orientations, compositions, and hierarchical structures ranging from the nanoscale to the microscale.^5^, ^6^, ^7^ In the last two decades, there have been increasing research efforts focused on the production of new functional structures based on peptides and their derivatives, either by copying the functions of peptides and proteins or by using totally new design rules. The resulting nanoscale materials are expected to have many applications, including as in biomedical materials and nanotechnology.^8^ Peptide materials have been successfully employed as fundamental components in biological membranes, nanodevices, hydrogels for cell culture and drug delivery, biosensors, functional materials with unique biorecognition abilities, and energy‐conversion materials. All of these applications of peptide‐based materials can be attributed to the characteristic structural/chemical features of the materials, which enable their functioning in an extremely wide context of fundamental and applied sciences.^9^

The field of peptide‐based materials was established in the early 1990s when Zhang et al. discovered the sequence of the ionic‐complementary peptide EAK16 (E: glutamic acid, K: lysine, A: alanine), which can assemble into interwoven filaments.^10^ Ion pairs present in the same chain simultaneously form hydrophilic faces, while alanine residues form a hydrophobic face, thus leading to a β‐sheet structure. A planar cyclic peptide that can stack and assemble into a nanotube with an internal diameter of ≈1 nm was previously investigated.^11^ The cyclic peptide with its even number of alternating d‐ and l‐amino acids can stably adopt a flat ring‐shaped conformation with a β‐sheet secondary structure. In 2001, Stupp and co‐workers explored new assembling motifs called peptide amphiphiles, which have a hydrophilic amino acid sequence and a hydrophobic alkyl tail.^12^ The distinct hydrophobic interaction has enabled the use of a broad range of peptide sequences; most peptide amphiphiles self‐assemble into cylindrical nanofibers with a β‐sheet conformation. A very short assembling motif, diphenylalanine (FF), which can maximize the advantages of both easy synthesis and direct control of the assembled structures, was reported by Reches and Gazit.^13^ The aromatic FF sequences can organize into highly stable tubular nanostructures due to strong π–π interactions of the side chains with backbone hydrogen bonding. By tuning these interactions, the nanostructure and properties can be controlled, for example, in sol to gel transition.^14^, ^15^ In addition to the above peptides, a variety of assembling motifs, such as the Fmoc‐peptide,^16^ hairpin peptides,^17^ amyloid‐forming peptides,^18^ and surfactant‐like oligopeptides,^19^ have been studied. Their ability to form specific secondary structures provides attractive platforms for the design of nanomaterials with controllable structural features. In recent years, not only the structural advantages of peptide assembly but also the unique chemical/physical properties of peptide‐based materials have been discovered. For example, assembled peptide nanotubes acting as catalytic sites for retro‐aldol reactions and imine condensation with enantioselectivity,^20^ visible light‐emitting supramolecular assemblies based on peptide–DNA conjugated molecules,^21^ flexible and tough layered peptide crystals,^22^ and sequence‐specific chiral templates for single‐helical gold nanoparticle (NP) superstructures^23^ are recent discoveries. In this respect, peptide self‐assembly has tremendous potential as a practical approach for multifunctional interdisciplinary materials.

A number of peptide‐based building blocks have been designed and developed to create supramolecular structures.^24^ Among them, FF dipeptides have drawn much attention due to their unusual chemical and physical properties and their very short length that can endow expandability toward hybridization with other motifs. Studies of FF sequences are inspired by the key motif of amyloid‐β peptides that assemble to form amyloid plaques in the brains of Alzheimer's patients. Two consecutive phenylalanine residues in amyloid‐β peptides are known as the core recognition motif due to their strong hydrophobic interactions via π–π stacking. The stacking interactions of FF dipeptides induce the formation of highly stable nanostructures with remarkable mechanical, electrochemical, electronic, and photochemical properties in addition to high thermal and chemical robustness and environmental compatibility.^25^ Thus far, the majority of short peptide‐based materials have been centered on the self‐assembly of FF‐based peptide sequences into nanostructures such as fibers, tubes, vesicles, and hydrogels. Several excellent reviews outline the design aspects, self‐assembled morphologies, and applications of these systems.

Recently, our group and Lee and co‐workers discovered a new class of assembling motifs from tyrosine (Y)‐rich short peptides that can assemble into macroscopic 2D nanosheets.^26^ Being slightly different from phenylalanine with a benzene ring, tyrosine has a phenolic —OH group as a side‐chain functional group. Tyrosine is a versatile amino acid that plays an important role in regulating the structural conformation transitions of proteins; its redox‐active property also facilitates interactions between metal ions in the active centers of enzymes and the transport of electrons along with protons.^27^, ^28^ In addition, it was observed that tyrosine‐rich regions in all the possible 7‐mer sequence domains occurring in an amyloidogenic protein, i.e., β_2_‐microglobulin (β2m), show the highest assembling propensities.^29^ This result suggested that the tyrosine residue acts as a key motif in the self‐assembly process. Inspired by the fascinating characteristics of tyrosine, for the first time, we systematically constructed a peptide library composed of short peptides with repeating tyrosine units in 2014 to study their assembly behavior and potential applications. The exploration and evaluation of supramolecular structures based on tyrosine‐rich peptides with unique features are opening up new avenues for sequence‐specific peptide‐based functional materials. In this review, we will discuss the roles of the tyrosine motif in natural systems and then review several approaches to mimic or utilize the function of designed tyrosine‐based peptide sequences. The first section of this review will address the role of tyrosine in nature, especially in terms of its electrochemical, chemical, and structural properties. In the second section, several interesting biomimetic molecular designs based on the proton‐coupled electron transfer (PCET) mechanisms of tyrosine will be considered. Later, chemical cross‐linking between tyrosine residues to develop biomaterials will be discussed. We will analyze in detail tyrosine peptide‐based materials from self‐assembled structures to peptide/inorganic hybrid systems. Finally, a brief account of the opportunities and future challenges in the design optimization and applications of tyrosine peptide‐based biomimetic systems will be provided.

## 2 The Role of Tyrosine in Natural Systems

### 2.1 Tyrosine as a Redox Cofactor

Precisely controlled electron‐transfer processes are essential for all living organisms, and complex protein/peptide assemblies manipulate electron‐transfer reactions. In respiration, for instance, electron transfer across long distances between proteins or across a membrane plays an important role in creating an electrochemical proton gradient, which is involved in producing adenosine triphosphate. Such electron‐transfer reactions in nature are commonly coupled to proton‐translocation processes to facilitate efficient charge transport. The coupling of electron and proton transfers between donors and acceptors in proteins can avoid high‐energy intermediates; this process is known as proton‐coupled electron transfer. PCET was introduced in 1981 to explain the basic process by which electrons and protons are transported together in a disproportionation reaction between Ru^IV^(bpy)_2_(py)(O)^2+^ and Ru^II^(bpy)_2_(py)(OH_2_)^2+^.^30^ Since then, the PCET mechanism has been extensively used for all reactions and half‐reactions that carry electrons and protons together.

**Figure 1:**
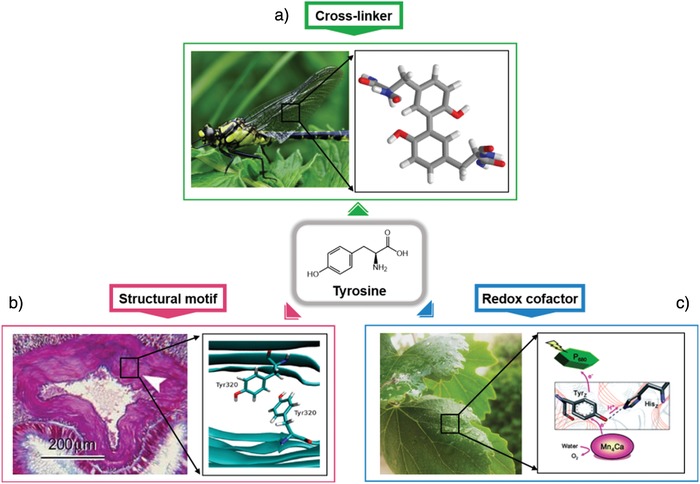
The role of tyrosine in natural systems. a) The presence of dityrosine bonds in resilin from the wings of a dragonfly. b) π–π interactions between tyrosine residues inducing amyloidogenic assembly. Reproduced with permission. Copyright 2017, American Chemical Society. c) PCET of tyrosine in photosystem 2 from chlorophyll. Reproduced with permission. Copyright 2011, Royal Society of Chemistry.

Redox cofactors embedded in a protein matrix are transiently oxidized and reduced and guide radical transport pathways.^31^ Several amino acids and their derivatives have been implicated in these processes, but the key player is the redox‐active amino acid tyrosine. Tyrosine with a redox‐active phenol group has been proposed to play an important role in several enzymes, including photosystem II (PS2),^32^, ^33^ ribonucleotide reductase,^34^ prostaglandin synthase,^35^ cytochrome c oxidase,^28^ and galactose oxidase.^36^ In the case of PS2, light‐induced charge separation reactions occur in the Mn_4_Ca cluster catalyst via a tyrosine denoted as Tyr_z_ (**Figure** **1**). It plays a critical role as a redox intermediate between the Mn_4_Ca cluster and the central chlorophylls, denoted as P_680_ from their lowest absorption maximum at 680 nm. Tyr_z_ transfers electrons from the Mn_4_Ca cluster to P_680_ in a steady‐state oxygen evolution process. When Tyr_z_ is oxidized, a neutral tyrosyl radical is formed, which accompanies the deprotonation of phenolic oxygen. A nearby histidine (His_z_) acts as a proton acceptor, hydrogen bonded to the phenolic proton. In other words, Tyr_z_ prevents the recombination of excited electrons and holes in P_680_ and efficiently transfers electrons from water molecules to P_680_. When the hydrogen bond between Tyr_z_ and His_z_ is destroyed by mutational replacement, the function of Tyr_z_ is severely compromised and often results in lost functionality. This phenomenon illustrates the importance of the PCET function of tyrosine. Inspired by the role of tyrosine in enzymatic systems and photosynthesis, numerous model studies have been conducted on structurally defined biomimetic peptides, such as a β‐hairpin Tyr‐containing peptide designed de novo^37^ and a Tyr‐conjugated metal–ligand complex.^38^

### 2.2 Dityrosine as a Cross‐Linker

The phenolic side chain of tyrosine and its unique chemical reactivity enable its involvement in many molecular interactions and biosynthetic transformations. This reactivity provides an efficient way to influence protein activity, including the ability to transfer electrons and to chemically graft phenol groups to form dityrosine. Such cross‐linking reactions can be readily accomplished by special enzyme reactions,^39^ interactions with redox‐active metal complexes,^40^, ^41^ radiolysis,^42^ or photolysis.^43^ The oxidative binding propensity of tyrosine residues has essential functions in organisms, but it also results in the pathogenic conjugation of proteins^43^, ^44^ (Figure 1). Such pathology is usually a part of the natural aging process, oxidative stress, and diverse pathological conditions. In biological systems, free dityrosine is not incorporated in de novo protein synthesis and has a high chemical stability (unreactive to changes in oxygen/pH). Due to these features, dityrosine has been used as an efficient biomarker (it has a characteristic fluorescence at 420 nm).^45^ Despite such potential detrimental influences, evolution has utilized this reaction to form a number of unique and highly specialized extracellular matrices that determine tissue structure and function.

Dityrosine chemical cross‐linking stabilizes the structure of proteins and strengthens the mechanical properties of several structural tissues. Resilin is a member of a broad family of elastic proteins that includes elastin, gluten, and gliadin; further, it induces low stiffness and energy‐storage domains in areas of the cuticles that experience high strains and rapid energy release.^46^, ^47^ The elasticity, mechanical properties, and energy‐storage properties of resilin were first investigated to determine the mechanism of flight of desert locusts and dragonflies.^48^, ^49^ Weis‐Fogh proposed a model in which randomly coiled protein chains were covalently cross‐linked and experienced a substantial reduction in entropy upon straining. The theoretically proposed chemical cross‐links were later discovered in dityrosine and trityrosine polypeptides.^47^ In addition to resilin, dityrosine residues have also been found in structural and connective tissues, such as in elastin from chick aorta,^50^ rabbit skin,^51^ human aorta,^52^ and bovine skin,^53^ and in silk fibroin from caddisfly and silkworm. The prevalence of naturally occurring dityrosine in structural proteins suggests that dityrosine is an important stabilizing motif and thus worth utilizing in the development of novel biomaterials. Therefore, there have been several attempts to introduce these mechanical and structural properties into materials engineering.^54^, ^55^, ^56^

### 2.3 Tyrosine as a Structural Motif

For protein folding and the determination of 3D architecture, specific sequence motifs and long‐range patterns of hydrophobic/hydrophilic moieties are extremely important. There have been reports on specific local arrangements of sequences that prefer certain secondary structures, e.g., residues that strongly prefer the *N*‐terminal and *N*+1 positions at the start of an α‐helix, general turn‐inducing sequences, and repeat positions of the heptad leucine, which favors a coiled‐coil or leucine zipper interaction. Of the 20 amino acids found in protein structures, tyrosine, phenylalanine, tryptophan, and histidine contain aromatic functional groups. The interactions between these aromatic residues are integral to the proper structure and function of proteins.^29^, ^57^ Recently, the single amino acid tyrosine has been shown to self‐assemble into cross‐β‐like fibers through hydrogen bonding and π–π stacking, and the fibrils were shown to mediate the aggregation of proteins through amyloid cross‐seeding, illustrating the importance of tyrosine residues in controlling the structure and function of proteins.^58^ Additionally, the sheet‐forming propensities of all possible 7‐mer sequence domains occurring in the amyloidogenic protein β2m were studied, and an important role was ascribed to the tyrosine‐rich regions.^59^ Interestingly, the sequences containing a large number of tyrosine residues exhibited a high Tango score, which is the score from a well‐validated algorithm used to analyze the propensity for amyloid‐assembly formation. Moreover, a tyrosine cross‐strand ladder influences the conformation and rigidity of multilayer stacking in β‐sheet‐forming proteins through π–π interactions between aromatic side chains.^60^ These observations indicate that tyrosine plays an important role in the self‐assembly process. Unlike phenylalanine, tyrosine has a phenolic —OH group that can undergo hydrogen bonding. In this context, various proteins, such as the Greek key β‐barrel protein, immunoglobulin, and fibronectin type III, contain a tyrosine‐containing local motif called a “tyrosine corner.” The tyrosine corner is a conformation in which a tyrosine near the beginning or end of an antiparallel β‐strand forms a hydrogen bond between its phenolic —OH group and the backbone amide of nearby tyrosine residues, resulting in the formation of nucleus folding and its stabilization.^27^, ^61^, ^62^, ^63^

## 3 Molecular Tyrosine as a Redox‐Active Linker

### 3.1 Proton‐Coupled Electron Transfer of Tyrosine

**Figure 2:**
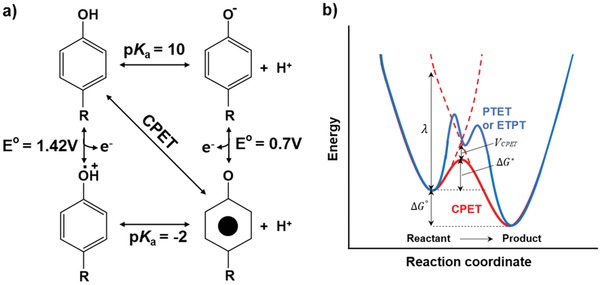
PCET system of tyrosine. a) Thermodynamic cycle of PCET for tyrosine radical formation. The vertical and horizontal pathways show stepwise ETPT and PTET mechanisms, while the diagonal pathway shows concerted proton and electron transfer directly from TyrOH to TyrO*. The CPET pathway avoids charged intermediates, which are advantageous in low dielectric media such as the interior of proteins. b) A conceptual reaction coordinate diagram for the PCET process. The CPET designated by the red line has a single transition state, while the PTET and ETPT processes designated in blue line have multiple transition states and high‐energy intermediate.

Among the various amino acids, tyrosine plays an essential role as a high‐potential redox‐active cofactor, for example, as a hopping site for long‐distance electron transfer. In particular, the phenolic group in tyrosine can mediate the PCET processes in many biological systems. The PCET process couples long‐distance electron transfer from the active centers of enzymes and short‐distance proton transfer to the surrounding bases. **Figure** **2** shows the thermodynamic cycle of the PCET process of tyrosine radical formation and the reaction diagram for both consecutive and concerted pathways. As shown in the figure, the p*K*_a_ value of every tyrosine oxidation pathway is in the range of 10 to −2, meaning that at most pH values, the oxidation of tyrosine is coupled to proton transfer.^64^

In general, PCET includes consecutive steps of proton transfer electron transfer (PTET) or vice versa (electron transfer proton transfer (ETPT)) and concerted proton and electron transfer (CPET). Among these, CPET is more effective due to its lower energy single‐step reaction, which avoids the formation of charged tyrosine species. Analogous to the electron‐transfer reaction, CPET can also be viewed as tunneling of electrons and protons at the same transition state, and the rate constant can be described as shown in Equation (1) in the same manner using the Marcus theory

(1) $$\begin{array}{l} k_{\mathrm{CPET}}=\;\;\frac{2\pi }{\hbar }V_{\mathrm{CPET}}^{2}{\left(4\pi \lambda RT\right)}^{-1/2}\exp\left(-\;\;\frac{{\left(\Delta G_{\mathrm{CPET}}^{0}+\lambda \right)}^{2}}{4\lambda RT}\right)\;\\ \mathrm{where}\;V_{\mathrm{CPET}}\approx V_{\mathrm{ET}}\times \;\;V_{\mathrm{PT}} \end{array}$$

In Equation (1), $\Delta G_{\mathrm{CPET}}^{0}$ is the free energy of the CPET reaction, and λ is the reorganization energy of the nuclear configuration to form the transition state from the equilibrium state in the reaction diagram (Figure 2b). Compared to pure electron transfer, every parameter is affected by the proton. For example, $V_{\mathrm{CPET}}^{}$ indicates the combined vibronic coupling of the CPET process, which is the product of electronic coupling, *V*_ET_, and proton coupling, *V*_PT_.^65^

Hydrogen bonding of tyrosine residues with surround residues is key to proton transfer in many biological systems, and the overlap between the donor and acceptor proton vibrational wavefunctions strongly depends on the proton‐transfer distance.^66^ Therefore, the mechanism of PCET strongly depends on noncovalent interactions in proteins that can modify the overall geometry.^67^

PCET reactions mediated by tyrosine exist in biological systems, such as in PS2 and blue‐light receptor proteins. In both cases, hydrogen bonding is key to determining the decay rate of tyrosine radicals, and if hydrogen bonding is either changed or disrupted, the nature of electron transfer changes significantly. In PS2, there are two redox‐active tyrosines, Y161(Tyr_z_) and Y160(Tyr_D_), in the D1 and D2 subunits. These tyrosines have two different surrounding protein environments, with two distinct roles in the electron transfer reaction. Y_Z_ acts as a redox intermediate to transfer electrons from the Mn_4_Ca cluster to P_680_+, while Y_D_ acts as a side‐path electron donor at night and does not participate in electron transfer when exposed to continuous light. Both of these oxidation reactions of tyrosine are coupled to proton transfer in a histidine residue. Due to the extensive hydrogen bonding in Tyr_z_, the electron transfer rate is much faster in Tyr_z_ than in Tyr_D_.^65^, ^67^

The importance of hydrogen bonding in tyrosine for the PCET process can also be observed in blue‐light receptor proteins. When excited by blue light, PCET can occur between tyrosine and flavin to form neutral flavin/tyrosine radical pairs. The reconfiguration of hydrogen bonds around flavin occurs under the action of surrounding light and alters the nature of PCET. In the dark‐adapted state, ETPT can occur where electron transfer is followed by proton transfer, while fast CPET occurs in the light‐adapted state due to the increasing basicity of nearby nitrogen atoms.^68^

### 3.2 Biomimetic Model Study of PCET

It is not easy to study the detailed mechanism of the PCET process in nature due to the complexity of large biological systems. However, a mechanistic insight into PCET can be a powerful tool for the effective design of powerful catalysts for water splitting and solar‐fuel generation by artificial photosynthesis.^66^ The hydrogen‐bond distance and geometry should also be carefully controlled to optimize reaction rates at a given reaction free energy.^64^ Therefore, diverse biomimetic models have been investigated as a more structurally well‐defined system to define a PCET reaction mechanism.^67^

**Figure 3:**
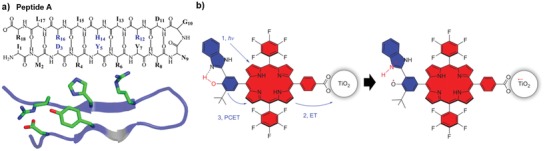
A biomimetic model system utilizing PCET. a) Structure of a β‐hairpin maquette peptide A: Backbone sequence and peptide folding induces π–π stacking of tyrosine and histidine and indirect hydrogen bonding with water molecules. Reproduced with permission.^67^ Copyright 2015, American Chemical Society. b) Artificial system *triad‐1* inspired by the high quantum efficiency water oxidation reaction in P_680_ in PSII. Upon irradiation, the system undergoes primary electron transfer and a PCET reaction. Reproduced with permission.^71^ Copyright 2014, Nature Publishing Group.

As shown in Figure 2, the formation and decay of neutral tyrosine radicals occur by a PCET process. At very high pH values, where tyrosine radicals decay via electron transfer, the decay rate is much faster than that at lower pH values. Both the surrounding pH and specific noncovalent interactions can control the tyrosine decay rate. The most well‐known model to study the effect of the protein environment on the tyrosine‐radical decay rate is the β‐hairpin maquette “Peptide A” model; its structure is described in **Figure** **3**a. Peptide A is a designed 18‐mer peptide inspired by PS2, and the p*K*_a_ of tyrosine in the strand is similar to that of tyrosine in solution. Due to peptide folding (Figure 3a), π–π stacking and hydrogen bonding of tyrosine and histidine occur, leading to a much faster orthogonal PCET for the decay of tyrosine radicals than for molecular tyrosine itself. This phenomenon suggests an increased electronic coupling due to noncovalent interactions between tyrosine and histidine in peptide A.^67^

Many biomimetic model studies on PCET use a simple platform, i.e., tyrosine attached to a photosensitizer, which is usually Ru(bpy)_3_^2+^. As the metal‐to‐ligand charge‐transfer excited state of Ru^2+^ is formed upon irradiation with 460 nm visible light, it can be oxidized in solution to form a Ru^3+^ intermediate. Due to the high reduction potential (1.26 V vs normal hydrogen electrode) of Ru^3+^, tyrosine can be oxidized to form tyrosine radicals via PCET. Several studies have used this platform to investigate the rate dependence of the reaction on factors such as intramolecular proton‐transfer distance using a variety of nearby bases, which can alter the nature of hydrogen bonds. All of these biomimetic models suggest that strong proton coupling and reduced reorganization energy due to strong hydrogen bonding and shorter proton‐transfer distances facilitate the PCET reaction to form tyrosine radicals; the rate can be dramatically changed by small geometric differences.^66^, ^69^

### 3.3 Artificial Photosynthesis Stimulated by Tyrosine

A significant number of artificial photosynthetic systems stimulated by the high quantum efficiency of water oxidation in nature have been developed to optimize the solar energy conversion process. Not surprisingly, the key component of these structures is a phenol moiety, which is also present in tyrosine and plays an essential role in the PCET process. Figure 3b shows an example of an artificial photosynthesis reaction center highlighting the practical application of the phenol moiety as a water‐oxidizing catalyst. This bioinspired triad‐1 consists of three covalently linked redox subunits and mimics both a short internal hydrogen bond and the thermal relaxation that accompanies PCET in PS2. Upon irradiation with porphyrin, electron transfer occurs in nanoparticles, such as SnO_2_ or TiO_2_, as shown in Figure 3b, whose dye radical cations are competent to P_680_^+^, and a second electron transfer from the phenol moiety of benzimidazole–phenol (BiP) to the porphyrin radical cation occurs. The incorporation of BiP and the formation of a neutral phenoxyl radical in the artificial system significantly improves the quantum yield for electron transfer to TiO_2_ nanoparticles.^70^, ^71^

In Section 3, we summarize the role of tyrosine as a redox‐active link. Inspired by the high quantum yield of the PCET electron‐transfer process mediated by tyrosine, a biomimetic model was analyzed with the long‐term goal of forming an experimental and theoretical platform for PCET processes. Moreover, for practically applying the lessons learned from the tyrosine PCET process, a number of research efforts on energy conversion equipment, such as solar fuel cells and electrochemical devices, are currently being undertaken. These recent advances in constructing artificial photosynthesis reaction centers inspired by tyrosine may guide more efficient catalyst design for energy production.

## 4 Biomaterials Developed by Dityrosine Cross‐Linking

### 4.1 Dityrosine Cross‐Linking

Another representative potential use of tyrosine is the exploitation of the oxidation chemistry of the phenolic side chain. The phenolic side chain of tyrosine can be oxidized to various forms, such as 3,4‐dihydroxyphenylalanine or eumelanin, or can participate in oxidative cross‐linking with another tyrosine residue, resulting in dityrosine bonds. Dityrosine cross‐linking is frequently found in many natural materials, such as resilin, silk, alginate, and collagen. Dityrosine cross‐linking in these natural materials endows the systems with enhanced structural properties, such as resilience and a high fatigue lifetime.

**Figure 4:**
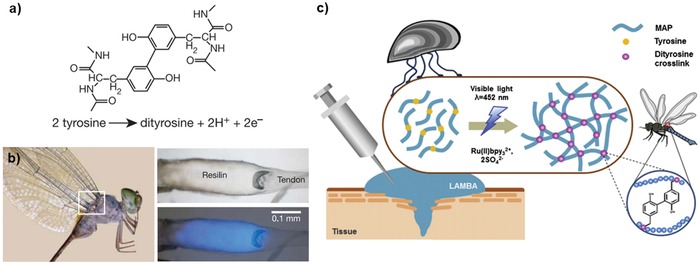
Dityrosine formation through cross‐linking. a) Chemical structure of dityrosine. b) Fluorescence of resilin in the wing tendon of an adult dragonfly. The right panels show photomicroscopic images of the tendon under white light (up) and ultraviolet light (down). Reproduced with permission.^72^ Copyright 2005, Nature Publishing Group. c) Schematic representation of the formation of a recombinant mussel adhesive protein‐based bioadhesive via dityrosine bonding using visible light. Reproduced with permission.^78^ Copyright 2015, Elsevier.

Resilin, which is found in the wings of dragonflies and the legs of fleas, is a representative example of dityrosine bonding, which resulted in increased structural stability (**Figure** **4**a). Elvin et al., in 2005, showed that cross‐linking between tyrosine residues in resilin can be quantitatively controlled through a photoinitiated process and that cross‐linking induced resilience in the protein, as measured by stress–strain measurements.^72^

There have been many developments of different methods to induce dityrosine cross‐linking. Enzymes, such as tyrosinase and peroxidase, have been used due to their selectivity with respect to side reactions; similarly, mild reaction conditions (pH and temperature) were used. The horseradish peroxidase enzyme contains a heme group with an Fe^3+^ core and is activated by hydrogen peroxide to form Fe^4+^ species, which then reacts with the phenolic side chain of tyrosine to form a tyrosine radical. The tyrosine radicals react with each other to form dityrosine bonds. Tyrosinase instead uses a complex of two copper atoms that can bind with phenolic —OH, which is then oxidized to diphenyl or quinone forms, thus forming tyrosine radicals that are cross‐linked with another tyrosine radical. The effectiveness of enzyme–base reactions is sometimes a drawback because the reaction is uncontrollably fast, and hence, the method has limited value for casting materials into shapes of desired dimensions.

On the other hand, many synthetic catalyst methods that promote cross‐linking, such as Fenton‐related reactions using peroxide interactions with iron ions or photoinitiated reactions using visible light with ruthenium tris‐bipyridyl ions as the catalyst, have been developed.^73^ The latter method has many applications in material design because it is driven by visible light, thus imparting versatility to the cross‐linking process in various systems. As a result, the ruthenium catalyst is widely used in the design of dityrosine‐based biomaterials.

With respect to photoactivation, tyrosine is oxidized upon UV irradiation; this oxidation may result in hydrogen abstraction, radical recombination, or isomerization and is a convenient method for the preparation of dityrosine without any additives, but the UV process can degrade other chromophores such as tryptophan or result in secondary products of tyrosine oxidation. The exact oxidation products vary from system to system; an analysis of these products is beyond the scope of this review, but it must be mentioned that in specific systems such as short peptides, UV irradiation is a strong tool to form tyrosine cross‐linking.^74^, ^75^

The formation of dityrosine bonds results in significant spectroscopic changes, which makes the detection of tyrosine cross‐linking a convenient task. Tyrosine has a characteristic absorption at ≈280 nm and fluorescence emission at ≈305 nm due to the presence of phenolic groups. After cross‐linking, the absorption shifts to ≈320 nm and the emission shifts to ≈410 nm.^76^ Although exact quantification is difficult, fluorescence emission from dityrosine bonds is deemed to be nondestructive and can be conveniently detected in various situations, such as in situ conditions; further, these emissions may be used to determine the degree of cross‐linking.

### 4.2 Material Design Using Dityrosine Cross‐Linking

Many material scientists have utilized dityrosine cross‐linking to develop materials for different applications; in most of these materials, improving the mechanical properties was the priority. This approach is actively used for peptide‐based hydrogels, where dityrosine can participate as a gelation factor by itself or as a way to increase mechanical stability and functionality.

Fang et al., through photochemical cross‐linking of a ferredoxin‐like protein, constructed hydrogels with good mechanical properties and a unique negative swelling behavior.^77^ The method can also be utilized for short peptide‐based systems. Ding et al. designed a peptide containing tyrosine residues with gel‐forming motifs and showed that tyrosine cross‐linking resulted in increased mechanical stability.^54^ Conjugation with protein derivatives leads to more applications and properties. Jeon et al. designed a light‐activated mussel protein‐based hydrogel that can be cross‐linked with controllable physical properties^78^ (Figure 4b).

The application of dityrosine cross‐linking is not limited to pure peptides but can also be extended to composite biopolymers. For example, chitosan and hyaluronic acids can be conjugated with tyramine, a derivative of tyrosine, to form cross‐links, resulting in hydrogels with synergetic properties, such as in vivo gelation or tissue adhesion.^79^, ^80^

Many different peptide sequences are known to participate in unique self‐assemblies, often with ordering at the molecular level. Dityrosine cross‐linking can lead to covalent bonds between the self‐assembled residues, resulting in stability and functionality without destroying the orderliness of the system. Min et al. showed that the tyrosine‐rich short peptide YYAYY can undergo cross‐linking when subjected to UV irradiation in different solvents, resulting in different nanostructures—a nanocapsule in aqueous media and lamellae in methanol.^81^ They also showed that YYAYY can undergo photochemical cross‐linking in the presence of a ruthenium catalyst, resulting in nanogels that can act as templates for biomineralization through UV irradiation with metal ions.^82^ Another usage of dityrosine cross‐linking is that it can be used for peptides in a self‐assembled state. Jan and co‐workers exploited the inherent nature of amphiphilic peptides to create self‐assembled micelles, which were then cross‐linked through UV irradiation, forming vesicles that are functionally applicable in various biomedical fields.^83^

## 5 Self‐Assembly of Short Tyrosine‐Rich Peptides

### 5.1 Macroscale 2D Assembly at the Air/Water Interface

The construction of atomically defined nanoscale structures from their components represents an important challenge for the development of advanced materials. In this regard, the advantageous features of peptide assembly are becoming more prominent in that the desired bulk properties of the resultant materials can be intricately adjusted by controlling the individual building blocks. Various peptides and their derivatives have been shown to have interesting hierarchical architectures, including tubes, fibers, planes, ribbons, and 3D networks.^5^, ^84^, ^85^ The design principles rely mainly on controlling the amphiphilicity, sequence periodicity, aromatic interaction, and charge distribution in the assembling units. However, identifying the key amino acids for a designed assembly is a goal that is made even more difficult by structural chirality and chemical complexity. In 2014, for the first time, our group and Lee and co‐workers systematically incorporated multiple tyrosine units into peptides of various lengths (2–7 amino acids) as a design element for the construction of a short‐peptide library to investigate the impact of tyrosine residues on self‐assembly.^26^ Additionally, cysteine was also inserted to confer folding stability through disulfide bridges. These peptides were self‐assembled into supramolecular structures with different morphologies. Most of the screened sequences formed 1D fibrillar structures in aqueous solutions. However, the YYACAYY sequence can assemble into nanosheets in solution. Interestingly, it was found that a facet was formed at the air/water interface of the water droplet containing the YYACAYY peptide.

**Figure 5:**
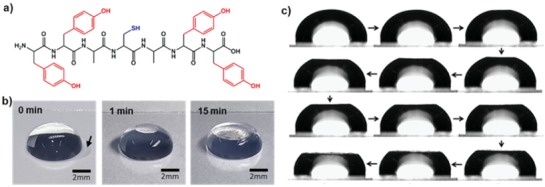
Facet formation on water droplets by 2D peptide sheets. a) Chemical structure of the YYACAYY peptide. b) Optical images of the water droplet with the passage of time. Reproduced with permission.^86^ Copyright 2016, American Chemical Society. c) Optical microscopy images of a water droplet at different incubation times. The shape evolution of the droplet was monitored using the side view. Reproduced with permission.^26^ Copyright 2014, Nature Publishing Group.

**Figure 6:**
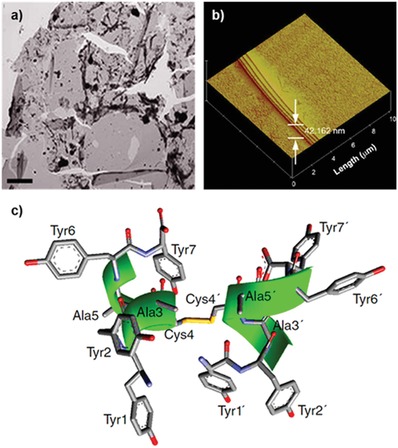
Structural analysis of the 2D assembly. a) TEM and b) AFM images of macroscale 2D sheets. c) Lowest‐energy NMR structure of the YYACAYY dimer. Reproduced with permission.^26^ Copyright 2014, Nature Publishing Group.

The faceting of water droplets could be seen with the naked eye, and their dynamic behavior was monitored using an optical camera (**Figure** **5**). Immediately after a water droplet containing YYACAYY was placed on a hydrophobic siliconized glass slide, a very thin transparent film was formed on the entire surface of the droplet within a few seconds. Subsequently, the top of the droplet was continuously flattened by the rigid sheets. The sheets could be easily transferred to a carbon grid or a silicon substrate for structural analysis by stamping the upper side of the faceted droplet. Transmission electron microscopy (TEM) showed the presence of 2D nanostructures, which grew in the lateral *x*–*y* dimensions (**Figure** **6**). Atomic force microscopy (AFM) analysis revealed that the assembled sheets were macroscopically homogeneous and comprised of multiple stacked nanosheets with a height of 1.4 nm, which was further confirmed by X‐ray diffraction studies. Flattening of water droplets requires very high mechanical energy, which indicates that the peptide sheet was mechanically strong enough to overcome the surface pressure of the droplet. The researchers calculated the elastic modulus of the peptide sheet using finite element analysis by counterbalancing the deformation energy of the final shape of the flattened droplet. The calculated elastic modulus was larger than 8.0 GPa, and nanoindentation analysis of the transferred peptide sheets showed that the measured elastic modulus was 8.4 GPa, which indicates that the peptide sheet is stiffer than cancellous bones.

Disulfide bridging by cysteine groups in YYACAYY was essential for the 2D assembly process. Both electrospray ionization (ESI) mass analysis and Raman spectroscopy analysis showed that the dimerization of YYACAYY occurred during sheet assembly, implying that the building blocks of the assembly process are disulfide‐bonded dimers. The molecular structure of the dimer was determined by 2D nuclear magnetic resonance (NMR). The conformational change of YYACAYY from a monomer to a dimer was monitored, and it was found that the dimer adopts a right‐handed α‐helical secondary structure, whereas the monomer remains unfolded. As shown in Figure 6c, eight phenolic tyrosine groups were facing the solvent. Additionally, the height of the dimer was ≈1.4 nm, which is equal to the distance between Tyr2 and Tyr6′; this value was close to the thickness of the sheet observed by AFM. The results also suggested that the dimers acted as building blocks.

In this context, Nam and co‐workers further expanded peptide sequences to induce facet formation using a macroscopic 2D assembly process and investigated in detail the assembly mechanism and structural arrangement. They demonstrated that the YFCFY sequence can induce facet formation at the air/water interface, and its assembly kinetics were significantly dependent on the ratio of the disulfide‐bonded dimer and monomer peptide.^86^ One interesting observation is that the YFCFY monomer assembled into fibril‐aggregated rough films, while YFCFY dimers formed uniformly packed flat nanosheets. The molecular conformation of the assembled structures and solvated peptide were analyzed by solid circular dichroism (CD) and 2D NMR, respectively. Nanosheets assembled on the water droplet were directly transferred to a quartz substrate, and solid CD analysis was conducted. Both solid CD and 2D NMR analyses clearly showed that the YFCFY dimer forms a helical conformation. These results indicated that the tyrosine‐containing building blocks of the interfacial 2D assembly adopt a helical secondary structure, which is further stabilized by disulfide bonds.

The discovery of laterally extended and untwisted 2D assembled structures is fundamentally difficult in peptide‐based systems. Due to the intrinsic chirality of peptides, the peptide backbone is mainly represented by only a single rotational state, resulting in a twist in each strand of the peptide.^87^ Macroscale flatness of the assembled structure constructed by the β‐sheet motif is also restrained by the propeller‐like twist in each β‐strand. Even sheet‐forming sequences can only assemble into tape‐like structures without being laterally extended over a few tens of micrometers.^88^ However, these studies provide a new strategy for macroscopic peptide 2D assembly via the stabilization of helical building blocks with tyrosine‐rich units and disulfide bonds.

### 5.2 Self‐Assembled Nanostructures by Tyrosine Cross‐Linking

The unique features of tyrosine–tyrosine crosslink networks found in natural enzymes offer several opportunities to impart structural integrity to supramolecular assembled structures. Kim and co‐workers presented a single‐step covalent self‐assembly approach with tyrosine‐rich short peptides using UV irradiation.^81^ They adopted the YYAYY sequence with symmetrical tyrosine pairs at both ends to achieve a high density of covalent bonds with neighboring tyrosines in proximate peptides. The morphology of the supramolecular assembled nanostructures was modulated by the selection of the reaction medium. For the synthesis of nanocapsules, YYAYY dissolved in a buffer (pH 10) was irradiated with UV light for 20 h. The average diameter of the assembled hollow capsules was 155.1 ± 46.13 nm, and the thickness of the shell was 2.5 ± 1.3 nm, as analyzed by dynamic light scattering and TEM, respectively. When the reaction medium was changed to methanol with 0.1 m NaOH, YYAYY formed free‐standing thin films after UV treatment. The assembled films were wrinkle‐free, several micrometers long along the lateral direction, and 4 nm thick. Cross‐linking and dityrosine formation were investigated using various spectroscopic and imaging techniques. As explained in the previous section, dityrosine bonds are known to exhibit absorption at ≈330 nm and emission at ≈410 nm. When the solution containing YYAYY was irradiated with UV light, the absorption intensity at ≈330 nm and blue fluorescence at 410 nm gradually increased. It is noteworthy that the formation of tyrosyl radicals increases with an increase in the pH; tyrosine cross‐linking upon UV irradiation occurs only after the deprotonation of phenol residues (p*K*_a_ ≈ 10.0).^75^ The mechanical properties of this covalently self‐assembled film were investigated through nanoindentation measurements. The measured elastic modulus of ≈30 GPa is higher than the previously reported values for tyrosine‐mediated assembled nanosheets and several biological proteinaceous materials.^89^, ^90^, ^91^ The peptide films retained their structure ever after exposure to harsh acidic conditions of 1 m HCl for a day. Such chemical and mechanical stability of the assembled structure can be attributed to the dityrosine cross‐links, which allow the protein to be mechanically stable, elastic, and resistant to proteolysis in natural systems.

**Figure 7:**
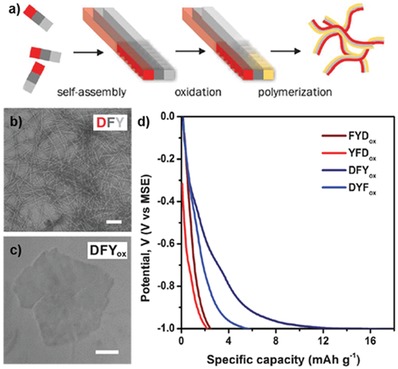
Sequence‐dependent polymeric peptide assembly. a) Schematic representation of the controlled formation of polymeric peptide pigments by enzymatic oxidation and further polymerization of preorganized tripeptides. TEM images of structures formed by the DFY peptide b) before enzymatic oxidation and c) after the reaction. d) Electrochemical potential profiles of the polymeric peptide pigments. Reproduced with permission.^92^ Copyright 2017, American Association for the Advancement of Science.

In addition to the UV cross‐linking chemistry, the enzymatic catalysis of tyrosine leads to not only dityrosine formation but also polymerization into melanin‐like supramolecular materials. Recently, Lampel and co‐workers reported the use of tyrosine‐containing tripeptides as tunable precursors for self‐assembly and synthesis of polymeric materials^92^ (**Figure** **7**). They first designed tripeptides composed of tyrosine, phenylalanine, and aspartic acid (d) to investigate the sequence‐dependent assembly behavior. After heating the aqueous solution containing each peptide and subsequent cooling to room temperature, six peptides show distinctive assembled structures, such as needle‐like crystalline fibers, nanofibrils, and amorphous aggregates. Later, they directly introduced tyrosinase, which can oxidize tyrosine into eumelanin, into the assembled structures for the synthesis of polymeric pigments.^93^ Interestingly, color changes occurred in all the tripeptides, and the products show very different colors, suggesting that the degree of polymerization varies greatly depending on the sequence and morphology. Most of the resulting polymeric materials maintained their own structural frame (e.g., fibers and amorphous aggregates), but their size and surface morphology underwent a change. However, as shown in Figure 7b,c, the DFY sequence first assembled into a dense network of nanofibrils, while DFY_ox_ (after enzyme treatment) polymerized into extended 2D sheets. Based on single‐crystal X‐ray diffraction analysis, the researchers proposed that the anticonformation of aromatic side chains is favorable for enzymatic polymerization along the length of the β‐sheet and laterally between nearby fibrils, leading to the destruction of fibrils and the formation of extended 2D sheets. The oxidized peptides displayed variable electrochemical properties originating from the tyrosine moiety. The charge‐storage capacity of each oxidized peptide was analyzed by electrochemical measurements to estimate the concentration of redox‐active components. DFY_ox_ 2D sheets showed the highest specific charge‐storage capacity, and this value was comparable to that of natural eumelanin; this observation can be ascribed to an increase in the concentration of redox‐active tyrosine‐based derivatives.

## 6 Tyrosine‐Rich Peptide‐Based Hybrid Functional Materials

Due to their attractive properties, especially functional flexibility and atomically defined nanostructures, peptides with specific sequences are gradually being designed for the development of new biomaterials. To enrich the applications of peptide materials and promote synergism between inorganic and peptide systems, several hybrid materials have been developed.^94^, ^95^, ^96^ In this section of the review, we will focus on tyrosine‐rich peptide‐based hybrid systems that can utilize the redox activity and π–π interactions of tyrosine to synthesize functional materials for catalysis and energy applications.

### 6.1 Self‐Assembling Peptide Scaffolds for Catalysis and Biomineralization

**Figure 8:**
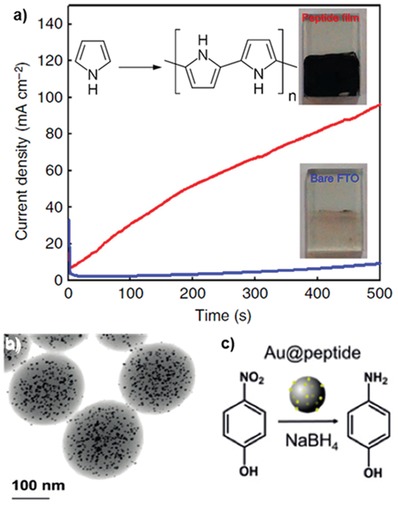
Self‐assembling peptide scaffolds for catalysis. a) The current density profiles of the YYACAYY peptide sheet (red) and the tyrosine monomer (blue) deposited on FTO substrates during bulk electrolysis at 0.9 V in a 0.1 m NaCl‐containing 50 × 10^−3^ m pyrrole solution. After 30 min of electrolysis, remarkably different kinetics of polypyrrole formation could be confirmed by the color changes of the substrate (inset). Reproduced with permission.^26^ Copyright 2014, Nature Publishing Group. b) Representative STEM image of the AuNP and YYAYY peptide hybrid nanogels. c) Catalytic reduction of *p*‐nitrophenol to *p*‐aminophenol by the hybrid nanogel. Reproduced with permission.^82^ Copyright 2018, John Wiley & Sons, Inc.

One potential application of tyrosine‐containing peptide assembly is to use it as a redox‐active scaffold for enzyme mimetic catalysts. Our group and Lee and co‐workers explored the possibility of exploiting tyrosyl radicals and YYACAYY nanosheets for pyrrole polymerization.^26^ At first, the in situ generation of tyrosyl radicals in peptide sheets was confirmed by the oxidation peak observed in cyclic voltammetry curves. Generally, pyrrole polymerization can be initiated by applying a potential of ≈0.9 V, but it requires a much higher potential to induce the macroscale deposition of polypyrrole.^97^ As shown in **Figure** **8**a, when a potential of 0.9 V was applied constantly to the sheets deposited on a fluorine‐doped tin oxide (FTO) substrate and bare FTO substrate, the current density of the peptide sheet/FTO sample was significantly higher than in the case of pure FTO, and macroscale black polypyrrole could be synthesized. Furthermore, it was demonstrated that treating the YYACAYY sheet with copper(II) ions could turn it into a pyrrole‐polymerization catalyst without the need for any applied electrical potential. However, solutions of copper(II) with tyrosine monomers could not produce polypyrrole. These results indicate that the assembled YYACAYY sheets with metal ions play a critical role in pyrrole oxidation.

Tyrosine usually enables the reduction of metal‐ion precursors due to the high redox potential of the phenolic group; further, the hydroxyl groups in phenol can also interact with metal ions.^98^, ^99^, ^100^ These interactions were utilized to construct metal–peptide hybrid structures through biomineralization. Kim and co‐workers developed semipermeable fluorescent peptide nanogels with a high density of tyrosine for a nano‐bioreactor, which allows the formation of uniform metal–peptide hybrids.^82^ The peptide nanogel was prepared using the YYAYY sequence exposed to UV irradiation to induce dityrosine cross‐linking. The average diameter and surface morphology of the nanogel were controlled by controlling the concentration of the peptide monomer and irradiation time. Metal–peptide hybrid nanogels were easily prepared by dispersing the nanogels into an aqueous AuCl_3_ solution. Scanning transmission electron microscopy (STEM) analysis clearly showed that discrete and uniformly sized gold nanoparticles were scattered in the nanogel without aggregation (Figure 8b). Additionally, the hybrid nanogel exhibited catalytic ability to reduce *p*‐nitrophenol to *p*‐aminophenol (Figure 8c). In a similar vein, Paribok et al. reported that silver nitrate can be reduced to silver nanoparticles (AgNPs) without any chemical reductant on tyrosine‐rich peptide nanofilms fabricated by the Langmuir–Blodgett or Langmuir–Schaefer deposition methods.^101^

### 6.2 Tyrosine‐Rich Peptide‐Based Hybrid Materials for Energy Applications

**Figure 9:**
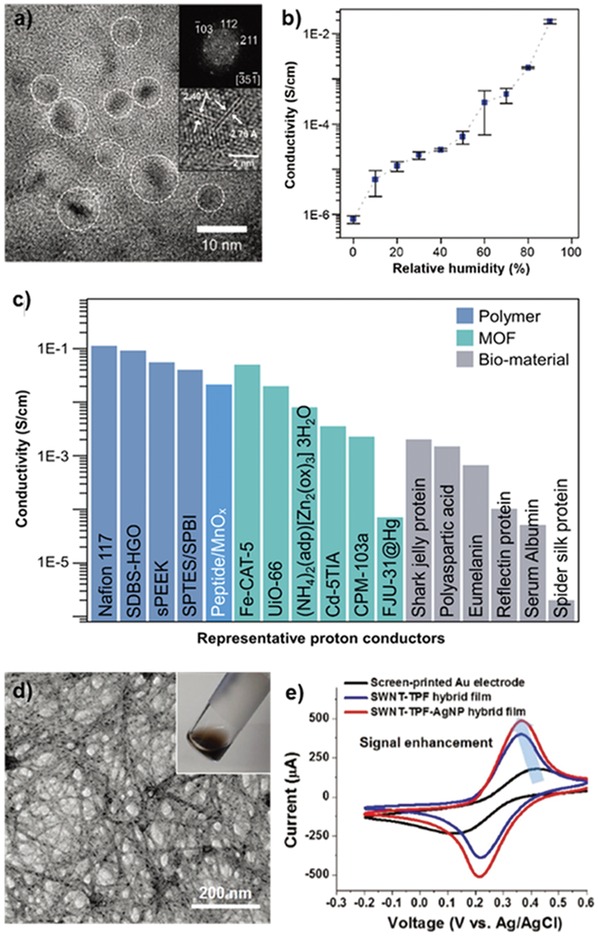
Tyrosine‐rich peptide‐based hybrid materials for energy applications. a) High‐resolution TEM images of peptide/manganese oxide hybrid nanofilms. b) Calculated conductivity of the hybrid nanofilm based on EIS analysis, depending on relative humidity. c) Comparison chart of the proton conductivity of other representative proton‐conducting materials, polymers, metal–organic frameworks (MOFs), and biomaterials at room temperature. Reproduced with permission.^102^ Copyright 2017, John Wiley & Sons, Inc. d) TEM image and photograph of a nanomesh assembly of SWNT–TPF–AgNP. e) Cyclic voltammetry curves of SWNT–TPF–AgNP, SNWT–TPF, and a bare gold electrode in a standard redox reagent, 10^−2^ m K_3_[Fe(CN)_6_]. Reproduced with permission.^106^ Copyright 2018, John Wiley & Sons, Inc.

In natural systems, several amino acids play an active role in charge transportation. The phenolic groups of tyrosine have been known to facilitate proton and electron transfer, interacting with metal clusters at the reaction center. Additionally, tyrosine can be oxidized into eumelanin with a proton/electron mixed conductivity, depending on the ambient humidity. To exploit the attractive features of tyrosine, Nam and co‐workers developed peptide/manganese oxide hybrid nanofilms for proton conductors.^102^ The spin‐coated YYACAYY peptide film was immersed in an aqueous KMnO_4_ solution. The high valence state of Mn can induce tyrosine oxidation and cross‐linking, resulting in the formation of manganese oxide. As shown in **Figure** **9**a, crystalline nanosized manganese oxide particles were embedded in the peptide film. To investigate the electrical behavior of the hybrid film, current–voltage measurements and electrochemical impedance analysis were conducted while varying the relative humidity (RH). Interestingly, the conductivity of the hybrid film increased as the surroundings became more humid (Figure 9b). Transient current measurements and *H*/*D* kinetic isotope experiments confirmed that the major charge carriers of the total current were protons. The calculated proton conductivity at 90% RH was ≈2 mS cm^−1^, which is much higher than that of pure manganese oxide or the peptide film, suggesting that there is a synergistic effect between the peptide and manganese oxide with respect to proton conduction. Moreover, the conductivity of the hybrid film is superior relative to the reported proton conductivities of biomaterials and comparable to that of other synthetic proton‐conducting materials (Figure 9c).

The ability of tyrosine to reduce metal ions, synthesize inorganic nanomaterials, and strongly interact with sp^2^ carbons through π–π interactions provides a great avenue for developing tyrosine‐based hybrid materials. In particular, the assembly of carbon nanoelectrodes with biological components is considered attractive for sensor applications.^103^, ^104^, ^105^ Self‐assembled peptide nanostructures can offer good templates with nondestructive and specific interactions with the target materials by tuning the functional group of the sequence. To fabricate nanoelectrodes containing carbon nanotubes and silver nanoparticles, self‐assembled tyrosine‐rich peptide nanofibers (TPF) were synthesized to function as a biological glue.^106^ TPFs were mixed with aqueous silver nitrate to directly decorate AgNPs on the surfaces of the nanofibers. Subsequently, the coupling of AgNP‐decorated nanofibers with single‐wall nanotubes (SWNTs) was carried out by simply mixing those two components in water with an anionic surfactant. During the dialysis process to eliminate unreacted residues, free‐standing composite SWNT–AgNP‐decorated films were formed; they were composed of an interweaved nanomesh network (Figure 9d). Furthermore, the use of these hybrid films for electrochemical sensor applications was investigated. As shown in Figure 9e, the electrochemical catalytic activity was screened using a standard redox molecule, K_3_[Fe(CN)_6_]. The oxidation and reduction currents of the redox molecule with the hybrid film were higher than those of a bare gold electrode and an only SWNT‐decorated film. These results can be attributed to the reduced tunneling length and increased surface area with active sites in the presence of grown AgNPs on the films. This particular study highlights new potential applications of tyrosine‐rich peptide‐based hybrid nanostructures as unique biomaterials for energy devices.

## 7 Conclusions and Outlook

From the practical perspective of nanomaterials, synthesizing materials with desired bulk properties and specific nanostructures is an ongoing challenge. Peptide self‐assembly, one of the most powerful bottom‐up approaches for the biomimetic construction of elaborate structures, is continuously expanding its range of application. Recently, a large number of studies on the synthesis and application of tyrosine‐rich peptide‐based systems have proven that tyrosine units serve as not only excellent assembly motifs but also multifunctional templates. The aromatic nature and redox‐active properties of the functional group act in a collaborative fashion, contributing to π–π interactions for the self‐assembly of the motif and charge transport via the PCET mechanism. As demonstrated by the examples discussed in this review, for the first time, our group introduced tyrosine‐rich short peptides as the building blocks of a macroscale 2D assembly and achieved the hybridization of functional inorganic components, which then synergistically enhanced the proton conduction ability. Ulijn and co‐workers demonstrated the ability to control the assembly and reactivity of tyrosine‐containing sequences to obtain polymeric pigments by enzymatic oxidation reaction. A variety of different supramolecular structures, including vesicles, nanofibrils, nanosheets, nanogels, and meshed networks, can be constructed with the self‐assembly and cross‐linking of tyrosine‐rich short peptides. Additionally, dityrosine chemistry can be widely applied to enhance the mechanical properties of biomaterials. Regarding future research on tyrosine‐rich peptide‐based building blocks, the ability to efficiently create functionalized 1D, 2D, and 3D peptide materials, supramolecular peptide–metal‐ion complex structures, and peptide–inorganic composite hybrid materials via spontaneous assembly will lead to numerous applications in membrane mimetics, device and sensor fabrication, nanoscale synthesis, and catalysis. These applications will collaboratively interconnect toward the development of a new family of robust and advanced biomaterials.

## Conflict of Interest

The authors declare no conflict of interest.
